# Developing a consensus-based motivational care pathway for individuals with lower limb fractures: a Delphi protocol

**DOI:** 10.3389/fpubh.2024.1384498

**Published:** 2024-07-16

**Authors:** Júlio Belo Fernandes, Sónia Fernandes, Ana Romão, Josefa Domingos, Rui Ferreira, Catarina Amador, Nelson Pardal, Domingos Malato, Ana Barroco, Ana Félix, António Oliveira, Fernanda Rito, Hélder Ratão, Rita Martins, Sandra Silva, Catarina Godinho

**Affiliations:** ^1^Egas Moniz Center for Interdisciplinary Research (CiiEM), Egas Moniz School of Health & Science, Almada, Portugal; ^2^Nurs* Lab, Almada, Portugal; ^3^Department of Nursing, Hospital Garcia de Orta, Almada, Portugal; ^4^Department of Nursing, Centro Hospitalar Barreiro Montijo, Barreiro, Portugal

**Keywords:** care pathway, fractures, bone, hip fractures, motivational interviewing, patient compliance, rehabilitation, exercise

## Abstract

Older adults with lower limb fractures often harbor concerns about losing their mobility, fearing a loss of independence. It is vital to develop strategies that foster their active engagement in the rehabilitation process. The present protocol aims to create a care pathway tailored to motivate individuals with lower limb fractures to adhere to rehabilitation. We will develop an observational, cross-sectional, and descriptive study using the Delphi data-gathering approach. Purposive sampling will recruit a panel of healthcare professionals and experts who care for patients with lower limb fractures. Aligned with the Delphi method, a series of iterative rounds will be developed to gather consensus around the motivational strategies used by health professionals in the rehabilitation of people with lower limb fractures. We will employ the Qualtrics platform for data collection and analysis, and a consensus target of 75% has been predetermined. For quantitative data analysis, we will use descriptive statistics encompassing a range of measures, including count, mean, standard deviation, median, minimum, maximum, and range. An inductive thematic analysis procedure will be employed to extract meaningful themes and patterns from qualitative data. The study results are expected to significantly impact clinical practice by creating a specialized care pathway to motivate individuals with lower limb fractures to adhere to rehabilitation. Adopting these explicit standards by professionals will ensure uniform and high-quality care.

## 1 Introduction

Bone fractures are a global public health concern, carrying a substantial economic impact on society (1). A bone fracture entails a complete or partial break in the anatomical continuity of bone caused by factors like high-force impact, stress, minor injuries, or specific medical conditions such as osteoporosis, resulting in a loss of mechanical stability (2, 3).

Fractures are not limited by age, affecting individuals of all age groups. However, the specific type and location of fractures differ significantly due to various factors, primarily attributed to individual bone quality and trauma (4). Bone fractures are prevalent and result in a significant financial burden on society due to their high healthcare expenses. Fractures pose a notable public health concern, leading to work absenteeism, decreased productivity, disability, health complications, and considerable healthcare costs. This substantially burdens individuals, families, societies, and healthcare systems (1, 5).

The incidence of fractures is rising (5–7), primarily attributed to the growing occurrence of fragility fractures among the expanding aging population (3). Lower limb fracture incidence globally differs due to factors including age, sex, socioeconomic status, and geography. Although comprehensive data is not available for every country, specific trends emerge (5). In 2019, over 178 million new fractures were reported worldwide, with patella, tibia, fibula, and ankle fractures being the most common, reaching around 32.7 million cases. Lower limb fractures accounted for 72.2 million incidents (5).

Significant progress have been made in medical and surgical approaches to ensure the rapid recovery of patients and alleviate rising healthcare costs (8). Following a lower limb fracture, patients often require physical therapy—a well-established approach in rehabilitating such cases (9) that yields enduring advantages such as enhanced physical function and diminished pain, resulting in an improved quality of life and alleviated healthcare system burdens as patients become empowered to manage their health (10–12). In addition, the rehabilitation team often recommends home exercise programs for clinical rehabilitation or self-managing long-term conditions (13). However, adherence to these programs remains challenging (14), impacting rehabilitation efficacy and potentially leading to the recurrence of injuries or reduced functionality (15).

Increasing adherence to rehabilitation is crucial, especially for older populations, as lower limb fractures can have a significant impact, even when rehabilitation is feasible. Previous studies have shown that older adults who experience a lower limb fracture, fear never regaining their ability to walk again and losing their independence (16–18). This underscores the need to develop strategies that actively motivate these individuals to engage in rehabilitation programs.

Prior studies show exercise self-efficacy correlates positively with initiating and maintaining exercise, especially in planned programs (19–22). However, a complex interplay of factors influences non-adherence to rehabilitation programs. Effective interventions must address a broad range of motivational constructs. According to Bandura’s Social Cognitive Theory, key constructs such as self-efficacy, outcome expectancies, observational learning, and self-regulation play critical roles in behavior change. Self-efficacy, or the belief in one’s ability to perform a specific task, is central to initiating and sustaining rehabilitation activities. Outcome expectancy, the belief that a particular behavior will lead to desired outcomes, further influences motivation (23, 24).

Interventions designed to motivate hold great promise (25–27). They can leverage these constructs by enhancing self-efficacy through mastery experiences (successful completion of tasks), vicarious experiences (observing others succeed), verbal persuasion (encouragement from healthcare providers), and managing physiological and emotional states to reduce anxiety and improve confidence (23, 24). By grounding interventions in Bandura’s theoretical framework, we can more effectively address the multifaceted nature of motivation. However, consensus on how to engage patients for fracture rehabilitation is still uncertain. The lack of guidance and formalized training may have a negative impact not only on patient experience but also on health outcomes. Therefore, this study aims to create a care pathway tailored to motivate individuals with lower limb fractures to adhere to rehabilitation.

## 2 Materials and methods

### 2.1 Study design

This research will employ an observational, cross-sectional, and descriptive design, utilizing the Delphi method as the data-gathering approach.

The Delphi method is widely recognized for achieving consensus and gathering expert opinions (28). Through a panel of experts, this study seeks to assess the extent of agreement and resolve disagreement on the motivational strategies used by health professionals. The Delphi method is well-suited to the study aims, allowing us to effectively engage numerous geographically dispersed experts cost-efficiently. Through this approach, we aim to develop an evidence-based care pathway that can enhance adherence and optimize recovery for individuals with lower limb fractures.

To ensure the quality of the research protocol report, we will follow the Recommendations for the Conducting and REporting of DElphi Studies (CREDES) (29).

### 2.2 Research steering group

A research steering committee will conduct and oversee this research endeavor. Their primary responsibility will involve formulating and disseminating the content for the Delphi rounds. The committee will comprise seasoned researchers with diverse expertise in general practice, geriatrics, nursing, and physiotherapy. Members of the research steering committee will oversee the surveys but not participate in them.

### 2.3 Participants and recruitment

The panel members’ experience and expertise will play a crucial role in maintaining the study quality (30). Therefore, we will use a purposive sampling approach to enhance the sample specificity and ensure that the outcomes remain relevant to the research context. This sampling approach allows researchers to select participants with distinct characteristics relevant to exploring data related to the subject of focus (31). We will purposefully select participants with diverse professions and ranges of caregiving experience for patients with lower limb fractures. This will enable the inclusion of participants who can provide diverse lived experiences. The study population includes healthcare professionals, such as nurses, doctors, and physiotherapists.

We will promote the study through various social media platforms such as Facebook, WhatsApp, and LinkedIn. Additionally, our research team will conduct thorough literature searches to identify national and international experts in the field. We will identify essential papers and extend invitations to the respective authors, formally requesting their participation on the panel. These invitations will be sent via email and social media. This approach ensures the formation of a diverse and knowledgeable group.

*Inclusion criteria*

- Be a healthcare professional/ researcher;
- Previous experience in caring for patients with lower limb fractures (2 year minimum);
- Proficiency in reading and understanding English;
- Willingness to participate in the study;
- Declaration of any conflicts of interest.

*Exclusion criteria*

- Insufficient experience in caring for patients with lower limb fractures (less than 2 years);
- Unable to commit to be available for the entire Delphi rounds.

The registration survey will ask participants about their profession and the number of years they have spent caring for others. This will facilitate the sampling process.

Appropriate candidates will be formally invited via email to join the panel, including comprehensive information regarding the study’s objectives, design, and commitment to participate in all Delphi rounds. The email will further encompass a brief online tutorial, lasting approximately 1–2 min, that expands on the overall Delphi process with visual support. This tutorial will aid in offering a clear understanding of the expected process. It will be emphasized that participation will extend over several months, encompassing multiple rounds of inquiries and feedback. At this stage, participants will also be invited to nominate peers interested in participating.

### 2.4 Data collection instrument

This study is built upon a prior scoping review (32) undertaken by the research team, which aimed to identify in the literature motivational strategies used by health professionals in the rehabilitation of people with lower limb fractures.

In light of the insights gained from the scoping review, the next phase of our research will involve developing a Delphi survey. This questionnaire will be constructed based on the motivational strategies that emerged from the scoping review (Table 1).

**Table 1:** Motivational strategies.

Strategies	Interventions
Therapeutic alliance	A thorough interview on admission.
	Develop a trusting and motivating relationship.
	Feelings of mutuality and respect in the alliance.
	Face-to-face counseling sessions.
Health literacy	Educate patients in rehabilitation exercise, complications, disease, and the benefits of exercise.
	Provide information leaflets/booklets.
	Cueing with posters describing the exercises.
Set achievable goals	Identify patients’ abilities and needs.
	Conferrer with patients to develop functional exercise goals at different stages of rehabilitation.
	Physical activity diary.
	Calendar of daily exercise activities.
Personalize the rehabilitation program	Develop an individually tailored exercise program.
	Tailor the instruction and program to make the task understandable.
Manage unpleasant sensations	Identifying challenges of postoperative rehabilitation through discussion.
	Use prescribed medications or heat/ice treatment to relieve or decrease pain.
Sharing cases	Share previous success stories to build confidence and motivate patients.
Problem-solving method	Identify obstacles to participating in the rehabilitation program.
	Use the problem-solving method to address perceived obstacles to participation in rehabilitation programs.
Persuasion	Describe the benefits of physical activities.
	Behavioral contract.
	Regular contact with patients via phone.
Encouragement and compliment	Assert that participants can self-manage.
	Provide positive verbal feedback upon their efforts.
	Give verbal encouragement and compliment.
	Motivation interviewing.
	Reinforce participants’ past and present successes or accomplishments.
	Family involvement.
	Digital activity coaching system.
Avoid negative emotional stimulation	Assess patients’ expressions of anxiety and depression.
Help to seek support	Telephone-assisted counseling.
	Identify individual barriers and resources for performing the exercise plan.
	Provide strategies for dealing with the identified barriers and coping in the future.

Critics have raised concerns about the limitations of traditional Delphi study designs in facilitating experts’ detailed explanations of their viewpoints (33). The present study will adopt a modified Delphi study design, as participants can elaborate on their opinions. In the initial round, they will also be prompted to identify any supplementary strategies they are familiar with or employ in their professional practice.

The survey will be developed using the Qualtrics platform (Qualtrics, Provo, UT, USA). Before it is applied, a pre-test will be conducted to assess comprehension and adequate functioning of the survey.

Each item will be evaluated using the Grading of Recommendations Assessment, Development, and Evaluation (GRADE) scale, which ranges from 1 to 9. The scale determines the significance of each item for inclusion: a score of 1–3 indicates it is not important for inclusion; 4–6 signifies importance, but not criticality; and 7–9 suggests it is critical for inclusion (34). The inclusion/exclusion criteria will consider items that achieve a ‘not important for inclusion’ or ‘critical-to-include’ rating in more than 75% of the responses. Given the absence of a clearly defined consensus criterion, we found this threshold and approach to be pragmatic and reasonable for consensus establishment, as they represents the perspective of three-quarters of the experts.

Items lacking consensus will progress to the subsequent round. Suppose there is no significant answer change about a specific item, characterized by a change of less than 20%. In that case, researchers will consider that consensus cannot be attained for that item and, therefore, exclude the item from subsequent rounds.

### 2.5 Rounds

**Figure 1:**
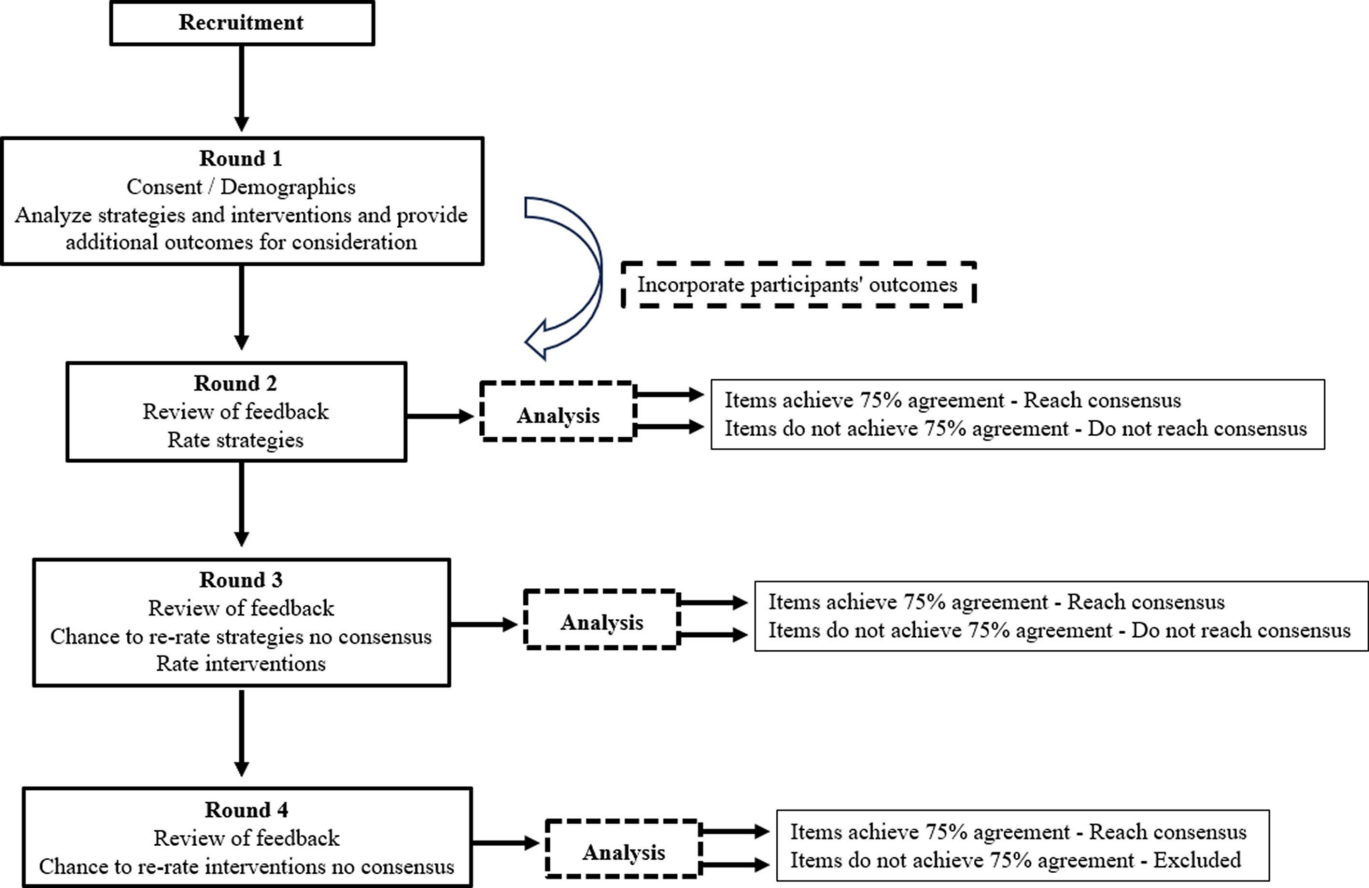
Flow chart to illustrate the stages of the Delphi process.

We will conduct a sequence of online survey rounds (Figure 1). Participants will receive exclusive links to each round via email on the first day. Each round is expected to take approximately 25–30 min to complete and will be open for 4 weeks. A minimum of 2 weeks will be allocated for result analysis between successive rounds. To ensure retention, participants will receive up to four email reminders for each round, encouraging completion before its closure, unless they choose to withdrawal from the study.

A total of 11 strategies have been identified for panel members to consider in Round 1 (Table 1). In Round 1, participants will access the online survey containing contextual details, survey instructions, and question-specific help resources. Demographic information will be collected, including age, sex, occupation, academic degree, years of work experience, and years of experience caring for patients with lower limb fractures. During this phase, participants will review the provided strategies and interventions, determining whether they wish to offer additional outcomes for consideration. Each survey item will feature a comment box where participants can suggest rephrasing, identify any omissions, propose new items, and offer reasons behind their choices.

The answers collected during each round will be aggregated and subjected to analysis. The summarized overview of data will be shared anonymously with participants as part of the introductory materials for the second round. This deliberate anonymity serves the dual purpose of fostering candid responses, as experts can contribute without being influenced by the input of their peers. Additionally, it empowers experts with the liberty to revise their initial responses if deemed suitable without any awareness on the part of their fellow experts. This confidentiality measure contributes to the integrity of the Delphi process and encourages genuine, uninfluenced participation.

In Round 2 participants will be asked to rate the appropriateness of the strategies as one that could be employed in patients with lower limb fractures. Following their ratings, participants will be asked to explain the reasons underlying their assessments.

In Round 3, the panel will have the chance to revisit their Round 2 responses for items where consensus is still pending. This opportunity arises from feedback on the group’s Round 2 responses. The overall process remains consistent with Round 2, albeit with a focus shift. Participants will be tasked with assessing the suitability of the interventions for potential implementation in patients with lower limb fractures. A fourth round will follow if consensus needs to be met after Round 3. During this round, panel members can reassess their Round 3 responses for items lacking consensus.

Like the previous round, participants will provide ratings for the appropriateness of these interventions. Following the rating process, participants will be encouraged to provide insights into the reasoning behind their evaluations.

Participants must respond to all rounds to successfully conclude the Delphi process. Consequently, individuals who did not participate in Round 2 will not be invited to participate in Round 3.

### 2.6 Panel size and composition

As this study employs qualitative research techniques, a predetermined minimum sample size was not calculated. Participants in a Delphi study are expected to possess experience and expertise relevant to the subject under investigation. There is no consensus regarding the appropriate number of experts, and no standardized criteria for determining the sample size have been established (35).

A faster consensus can often be achieved when using smaller panel sizes. However, a smaller panel may mostly represent a more homogenous group, limiting the validity of extending the findings to a broader population. On the other hand, a larger panel size allows for greater heterogeneity among the participants, making the results more relevant for a broader range of individuals. With an increase in panel size, the reliability of findings also improves, reducing errors (36). However, the authors propose that Delphi studies with panel sizes exceeding 25–30 experts do not yield new ideas or significant enhancements in outcomes (36, 37). Sample size recommendations for the consensus group (38) range from 20 to 24 panelists per group, considered sufficient to fulfill the study’s objectives, and numerous studies have incorporated even fewer than 20 participants (39–41). As such, our initial selection will encompass at least 95 experts, factoring in an estimated dropout rate of 25% between subsequent rounds of questioning (36).

### 2.7 Strategy to improve the response rate

The strategy to enhance the response rate will commence right from the outset of participant selection. The lead researcher (JBF) will contact each candidate to provide an overview of the study. Response rates will be boosted through a personalized approach, along with a clear explanation of the study process and an emphasis on the significance of their commitment to result validity (42).

After inclusion, participants will receive an email containing study details, supplemented by an email during the week preceding each round. The survey link will be emailed, and a text message will be sent to their mobile phones. To build rapport, regular email updates and social media announcements detailing study advancement will be disseminated throughout the process, including notifications about upcoming survey rounds.

Reminder emails will constitute a pivotal strategy to encourage the completion of each survey round, underlining the importance of their perspectives and stressing the necessity of completing the process for meaningful outcomes. When feasible, deadlines can be extended to accommodate participants’ schedules. Personalized reminder emails will be sent to establish a personal connection and motivate professionals to meet the deadlines. Participants will be informed about the number of completed rounds to foster a sense of achievement and inspire them to complete the round.

The lead researcher will facilitate communication with the panelists. Upon completing the survey, each participant will receive a personalized email expressing gratitude for their commitment to the project and survey completion.

### 2.8 Data analysis

The sample will be characterized using descriptive statistics involving various measures such as count, mean, standard deviation, median, minimum, maximum, and range. Subsequently, each item’s count, mean, minimum, and maximum scores will be calculated and reported to participants following each study round. To assess the stability of responses, we will employ measurement of central tendencies with dispersion and analyze the percentage and frequency of distribution within the group. These strategies will help us evaluate the consistency and reliability of responses throughout the Delphi study rounds.

The statistical analysis will be conducted using the IBM Statistic Package for the Social Sciences software (IBM Corp. Released 2020. IBM SPSS Statistics for Windows, Version 27.0. Armonk, NY, USA: IBM Corp.).

For the analysis of free-text responses, an inductive thematic analysis procedure, as described by Braun and Clarke (43), will be performed independently by two team members. The QDA Miner Lite software will support this process. This method will facilitate the identification of emerging themes from data through stages encompassing pre-analysis, encoding, categorization, and data interpretation.

### 2.9 Ethical considerations

The study will be conducted in accordance with the European Union General Data Protection Regulation and the Declaration of Helsinki (as revised in 2013). Therefore, the study protocol will be evaluated by an Ethical Review Committee. The initial page of the survey will feature a comprehensive elucidation of the study’s objectives and methodologies, accompanied by a guarantee that the researchers will ensure the confidentiality and anonymity of the data.

Participants must explicitly accept and agree to the online informed consent to proceed with the survey. The survey will have the options for participants to choose from “Yes” or “No,” indicating their understanding of the consent details and willingness to participate. Only those who affirmatively respond with a “Yes” to the informed consent query will be guided to the survey.

Participants who respond with “No” to the informed consent query will be guided to the survey’s conclusion, and their engagement will not be carried forward into subsequent rounds of the study. It will be entirely at the discretion of participants to determine whether to respond to any given question, modify their answers, or voluntarily quit at any time.

In strict adherence to ethical principles of anonymity and confidentiality, all data collected will be meticulously stripped of personally identifiable information, including any semblance of electronic identifiers.

All digital data will be coded, stored on a password-protected computer, and retained for 5 years. After this retention period, the lead researcher will destroy all data.

## 3 Discussion

The present study protocol outlines a Delphi study to establish a motivational care pathway tailored for individuals with lower limb fractures based on expert opinions and consensus. We will use the Delphi method to systematically develop a comprehensive care pathway that guides the rehabilitation journey.

There is a need to innovate and introduce new tools to enhance patient experiences and health outcomes (32, 44, 45). This proposed care pathway is a prime example of such a tool, aiming to address the unique challenges of individuals with lower limb fractures. Its development can be a step forward in providing tailored solutions that can markedly improve the patient’s journey toward recovery and well-being.

The results of this research are expected to significantly impact clinical practice and are directly relevant. Creating a specialized care pathway will establish explicit standards expected to be adopted by health professionals. This standardization can revolutionize the approach to patient care, facilitating a more holistic and patient-centric approach. In essence, the resultant care pathway aims to streamline the rehabilitation process, enhance patient outcomes, and ultimately contribute to raising the standards for care provision within this domain.

Applying the Delphi method in this study guarantees the anonymization of all individual contributions, thus maintaining an equitable weighting of each expert’s input (36, 37, 42). The deliberate inclusion of experts from diverse professional backgrounds aims to comprehensively represent the multifaceted stakeholders involved in healthcare delivery. This strategic inclusion holds the potential to foster wider acceptance and integration of research findings.

The forthcoming results of this study have the potential to bridge existing gaps within the literature. By expert consensus, a dedicated care pathway to motivate individuals with lower limb fractures will be developed. Remarkably, this will be the first care pathway designed for this intention.

Notably, the current landscape lacks well-defined motivational guidelines or recommendations tailored to these patients, even though evidence underscores the distressing reality that fractures can trigger concerns about future mobility in older adults and engender the perception that the fracture signifies the end of independent living (16–18). This accentuates the need to create a care pathway that motivates these individuals to participate in rehabilitation programs.

Establishing clear, consensus-based recommendations could potentially yield advantages for these patients. These include an improved patient experience, heightened adherence to rehabilitation programs, and, ultimately, better health outcomes. The fruition of such recommendations could mark a pivotal advancement in patient care, addressing emotional and psychological factors alongside physical recovery.

This study is not without limitations. First, there’s the potential for expert selection bias, which can inadvertently occur if professional backgrounds are unevenly represented within the expert panel. This might impact the comprehensiveness and diversity of insights gathered during the study. To mitigate this potential bias, we intend to employ purposive sampling techniques. Second, there’s a risk of potential groupthink, a phenomenon where the iterative nature of the method could inadvertently induce experts to gravitate toward consensus. To counteract this, we will anonymizing participant data, to diminish this risk, fostering an environment where participants feel comfortable expressing their opinions without apprehensions tied to social dynamics or hierarchy. This approach will stimulate participants and open contributions. Finally, the Delphi method requires a significant investment of time due to its iterative process spanning multiple rounds of data collection. This prolonged process might strain participant commitment and hinder the feasibility of completing all intended rounds. To minimize this risk, we anticipated a dropout rate of 25% over the consecutive rounds of consensus development. In addition, we have designed a series of strategies to improve the response rate.

## 4 Conclusion

This research protocol outlines a comprehensive approach to collecting expert opinions and achieving consensus through the Delphi method, to devise a motivational care pathway tailored for individuals with lower limb fractures. The findings can potentially fill gaps in the literature by guiding rehabilitation for individuals with evidence-based care pathways. The insights gathered with the development of this study have the potential to contribute significantly to improving patient care, offering tangible guidance for healthcare professionals to facilitate the recovery journey of individuals with lower limb fractures.

## Author contributions

JF: Writing – original draft, Writing – review & editing. SF: Writing – original draft, Writing – review & editing. AR: Writing – original draft, Writing – review & editing. JD: Writing – original draft, Writing – review & editing. RF: Writing – original draft, Writing – review & editing. CA: Writing – original draft, Writing – review & editing. NP: Writing – original draft, Writing – review & editing. DM: Writing – original draft, Writing – review & editing. AB: Writing – original draft, Writing – review & editing. AF: Writing – original draft, Writing – review & editing. AO: Writing – original draft, Writing – review & editing. FR: Writing – original draft, Writing – review & editing. HR: Writing – original draft, Writing – review & editing. RM: Writing – original draft, Writing – review & editing. SS: Writing – original draft, Writing – review & editing. CG: Writing – original draft, Writing – review & editing.

## References

[1] Polinder S Haagsma J Panneman M Scholten A Brugmans M Van Beeck E. The economic burden of injury: health care and productivity costs of injuries in the Netherlands. Accid Anal Prev. (2016) 93:92–100. doi: 10.1016/j.aap.2016.04.003 27177394

[2] Bigham-Sadegh A Oryan A. Basic concepts regarding fracture healing and the current options and future directions in managing bone fractures. Int Wound J. (2015) 12:238–47. doi: 10.1111/iwj.12231, PMID: 24618334 PMC7950494

[3] Hernlund E Svedbom A Ivergård M Compston J Cooper C Stenmark J . Osteoporosis in the European Union: medical management, epidemiology and economic burden. A report prepared in collaboration with the international osteoporosis foundation (IOF) and the European Federation of Pharmaceutical Industry Associations (EFPIA). Arch Osteoporos. (2013) 8:136. doi: 10.1007/s11657-013-0136-1, PMID: 24113837 PMC3880487

[4] Bergh C Wennergren D Moller M Brisby H. Fracture incidence in adults in relation to age and gender: a study of 27,169 fractures in the Swedish fracture register in a well-defined catchment area. PLoS One. (2020) 15:e0244291. doi: 10.1371/journal.pone.0244291, PMID: 33347485 PMC7751975

[5] GBD 2019 Mental Disorders Collaborators. Global, regional, and national burden of bone fractures in 204 countries and territories, 1990-2019: a systematic analysis from the global Burden of disease study 2019. Lancet Psychiatry. (2021) 9:137–50. doi: 10.1016/S2215-0366(21)00395-3 PMC8776563 35026139

[6] Abtahi S Driessen JHM Vestergaard P van den Bergh J Boonen A de Vries F . Secular trends in major osteoporotic fractures among 50+ adults in Denmark between 1995 and 2010. Osteoporosis Int. (2019) 30:2217–23. doi: 10.1007/s00198-019-05109-0, PMID: 31418061 PMC6811370

[7] Beerekamp MSH de Muinck Keizer RJO Schep NWL Ubbink DT Panneman MJM Goslings JC. Epidemiology of extremity fractures in the Netherlands. Injury. (2017) 48:1355–62. doi: 10.1016/j.injury.2017.04.047 28487101

[8] Einhorn TA Gerstenfeld LC. Fracture healing: mechanisms and interventions. Nat Rev Rheumatol. (2015) 11:45–54. doi: 10.1038/nrrheum.2014.164, PMID: 25266456 PMC4464690

[9] Perry SB Downey PA. Fracture risk and prevention: a multidimensional approach. Phys Ther. (2012) 92:164–78. doi: 10.2522/ptj.20100383, PMID: 21921251

[10] Koudouna S Evangelopoulos DS Sarantis M Chronopoulos E Dontas IA Pneumaticos S. The effect of postoperative physical therapy following hip fracture: a literature review. Cureus. (2023) 15:e37676. doi: 10.7759/cureus.37676, PMID: 37206486 PMC10189836

[11] Beckmann M Bruun-Olsen V Pripp AH Bergland A Smith T Heiberg KE. Effect of exercise interventions in the early phase to improve physical function after hip fracture - a systematic review and meta-analysis. Physiotherapy. (2020) 108:90–7. doi: 10.1016/j.physio.2020.04.009, PMID: 32726713

[12] Peek K Sanson-Fisher R Mackenzie L Carey M. Interventions to aid patient adherence to physiotherapist prescribed self-management strategies: a systematic review. Physiotherapy. (2016) 102:127–35. doi: 10.1016/j.physio.2015.10.003, PMID: 26821954

[13] Chen B Hu N Tan JH. Efficacy of home-based exercise programme on physical function after hip fracture: a systematic review and meta-analysis of randomised controlled trials. Int Wound J. (2020) 17:45–54. doi: 10.1111/iwj.13230, PMID: 31714005 PMC7948621

[14] Room J Boulton M Dawes H Archer K Barker K. Physiotherapists' perceptions of how patient adherence and non-adherence to recommended exercise for musculoskeletal conditions affects their practice: a qualitative study. Physiotherapy. (2021) 113:107–15. doi: 10.1016/j.physio.2021.06.001, PMID: 34571284

[15] Jack K McLean SM Moffett JK Gardiner E. Barriers to treatment adherence in physiotherapy outpatient clinics: a systematic review. Man Ther. (2010) 15:220–8. doi: 10.1016/j.math.2009.12.004, PMID: 20163979 PMC2923776

[16] Gesar B Hommel A Hedin H Baath C. Older patients' perception of their own capacity to regain pre-fracture function after hip fracture surgery - an explorative qualitative study. Int J Orthopaedic Trauma Nurs. (2017) 24:50–8. doi: 10.1016/j.ijotn.2016.04.005, PMID: 27554953

[17] Griffiths F Mason V Boardman F Dennick K Haywood K Achten J . Evaluating recovery following hip fracture: a qualitative interview study of what is important to patients. BMJ Open. (2015) 5:e005406. doi: 10.1136/bmjopen-2014-005406, PMID: 25564138 PMC4289715

[18] Hagsten B Svensson O Gardulf A. Early individualized postoperative occupational therapy training in 100 patients improves ADL after hip fracture: a randomized trial. Acta Orthop Scand. (2004) 75:177–83. doi: 10.1080/00016470412331294435, PMID: 15180233

[19] Fu W Li Y Liu Y Li D Wang G Liu Y . The influence of different physical exercise amounts on learning burnout in adolescents: the mediating effect of self-efficacy. Front Psychol. (2023) 14:1089570. doi: 10.3389/fpsyg.2023.1089570, PMID: 36891208 PMC9986600

[20] Isa T Ueda Y Nakamura R Misu S Ono R. Relationship between the intention-behavior gap and self-efficacy for physical activity during childhood. J Child Health Care. (2019) 23:79–86. doi: 10.1177/1367493518777297, PMID: 29783846

[21] Pedersen MM Zebis MK Langberg H Poulsen OM Mortensen OS Jensen JN . Influence of self-efficacy on compliance to workplace exercise. Int J Behav Med. (2013) 20:365–70. doi: 10.1007/s12529-012-9239-0, PMID: 22622819 PMC3767884

[22] Pekmezi D Jennings E Marcus BH. Evaluating and enhancing self-efficacy for physical activity. ACSMs Health Fit J. (2009) 13:16–21. doi: 10.1249/FIT.0b013e3181996571, PMID: 29910597 PMC6003667

[23] Bandura A Freeman WH Lightsey R. Self-efficacy: the exercise of control. J Cogn Psychother. (1999) 13:158–66. doi: 10.1891/0889-8391.13.2.158

[24] Bandura A In: Freeman WH, editor. Self-efficacy: the exercise of control. New York, USA: Henry Holt & Co (1997)

[25] Pudkasam S Feehan J Talevski J Vingrys K Polman R Chinlumprasert N . Motivational strategies to improve adherence to physical activity in breast cancer survivors: a systematic review and meta-analysis. Maturitas. (2021) 152:32–47. doi: 10.1016/j.maturitas.2021.06.008, PMID: 34674806

[26] Argent R Daly A Caulfield B. Patient involvement with home-based exercise programs: can connected health interventions influence adherence? JMIR Mhealth Uhealth. (2018) 6:e47. doi: 10.2196/mhealth.8518, PMID: 29496655 PMC5856927

[27] McGrane N Galvin R Cusack T Stokes E. Addition of motivational interventions to exercise and traditional physiotherapy: a review and meta-analysis. Physiotherapy. (2015) 101:1–12. doi: 10.1016/j.physio.2014.04.009, PMID: 25239472

[28] Humphrey-Murto S Varpio L Wood TJ Gonsalves C Ufholz LA Mascioli K . The use of the Delphi and other consensus group methods in medical education research: a review. Acad Med. (2017) 92:1491–8. doi: 10.1097/ACM.0000000000001812, PMID: 28678098

[29] Junger S Payne SA Brine J Radbruch L Brearley SG. Guidance on conducting and REporting DElphi studies (CREDES) in palliative care: recommendations based on a methodological systematic review. Palliat Med. (2017) 31:684–706. doi: 10.1177/0269216317690685, PMID: 28190381

[30] Powell C. The Delphi technique: myths and realities. J Adv Nurs. (2003) 41:376–82. doi: 10.1046/j.1365-2648.2003.02537.x, PMID: 12581103

[31] Malterud K Siersma VD Guassora AD. Sample size in qualitative interview studies: guided by information power. Qual Health Res. (2016) 26:1753–60. doi: 10.1177/1049732315617444 26613970

[32] Fernandes JB Vareta D Fernandes S Almeida AS Peças D Ferreira N . Rehabilitation workforce challenges to implement person-centered care. Int J Environ Res Public Health. (2022) 19:3199. doi: 10.3390/ijerph19063199, PMID: 35328886 PMC8950126

[33] Walker A Selfe J. The Delphi method: a useful tool for the allied health researcher. Br J Ther Rehabil. (1996) 3:677–81. doi: 10.12968/bjtr.1996.3.12.14731

[34] Guyatt GH Oxman AD Kunz R Atkins D Brozek J Vist G . GRADE guidelines: 2. Framing the question and deciding on important outcomes. J Clin Epidemiol. (2011) 64:395–400. doi: 10.1016/j.jclinepi.2010.09.012, PMID: 21194891

[35] Jandhyala R. Delphi, non-RAND modified Delphi, RAND/UCLA appropriateness method and a novel group awareness and consensus methodology for consensus measurement: a systematic literature review. Curr Med Res Opin. (2020) 36:1873–87. doi: 10.1080/03007995.2020.1816946, PMID: 32866051

[36] Chalmers J Armour M. The Delphi Technique In: Liamputtong P, editor. Handbook of research methods in health social sciences. Singapore: Springer Singapore (2019). 715–35.

[37] Nasa P Jain R Juneja D. Delphi methodology in healthcare research: how to decide its appropriateness. World J Methodol. (2021) 11:116–29. doi: 10.5662/wjm.v11.i4.116, PMID: 34322364 PMC8299905

[38] Trevelyan EG Robinson PN. Delphi methodology in health research: how to do it? Eur J Integr Med. (2015) 7:423–8. doi: 10.1016/j.eujim.2015.07.002

[39] Dang J Lal A Montgomery A Flurin L Litell J Gajic O . Developing DELPHI expert consensus rules for a digital twin model of acute stroke care in the neuro critical care unit. BMC Neurol. (2023) 23:161. doi: 10.1186/s12883-023-03192-9, PMID: 37085850 PMC10121414

[40] Veugelers R Gaakeer MI Patka P Huijsman R. Improving design choices in Delphi studies in medicine: the case of an exemplary physician multi-round panel study with 100% response. BMC Med Res Methodol. (2020) 20:156. doi: 10.1186/s12874-020-01029-4, PMID: 32539717 PMC7294633

[41] Marti-Martinez LM Gracia-Sanchez A Ferrer-Torregrosa J Lorca-Gutierrez R Garcia-Campos J Sanchez-Perez SP. Description of the surgical technique for condylectomy with minimally invasive surgery to treat interdigital helomas on the lesser toes: a Delphi study. J Foot Ankle Res. (2019) 12:13. doi: 10.1186/s13047-019-0322-5, PMID: 30815036 PMC6376773

[42] McKenna HP. The Delphi technique: a worthwhile research approach for nursing? J Adv Nurs. (1994) 19:1221–5. doi: 10.1111/j.1365-2648.1994.tb01207.x, PMID: 7930104

[43] Braun V Clarke V. Using thematic analysis in psychology. Qual Res Psychol. (2006) 3:77–101. doi: 10.1191/1478088706qp063oa

[44] Domingos J Dean J Fernandes JB Massano J Godinho C. Community exercise: a new tool for personalized Parkinson’s care or just an addition to formal care? Front Syst Neurosci. (2022) 16:916237. doi: 10.3389/fnsys.2022.916237, PMID: 35844246 PMC9280427

[45] Kelly CJ Young AJ. Promoting innovation in healthcare. Fut Healthc J. (2017) 4:121–5. doi: 10.7861/futurehosp.4-2-121, PMID: 31098448 PMC6502619

